# A Survey of Current Australasian Practices in the Use of Residual Bloodspots for the Addition of a New Disorder to the Screening Panel

**DOI:** 10.3390/ijns12020020

**Published:** 2026-03-30

**Authors:** Lawrence Greed, James Pitt, Ronda F. Greaves, Kate Coleman, Gabrielle Crisp, Enzo Ranieri, Mark de Hora, Dianne Webster, Natasha Heather

**Affiliations:** 1 Western Australia Newborn Bloodspot Screening Program, Department of Clinical Biochemistry, PathWest Laboratory Medicine WA, Perth 6008, Australia; 2 Victorian Clinical Genetics Services, Murdoch Children’s Research Institute, Parkville 3052, Australia; james.pitt@vcgs.org.au (J.P.); ronda.greaves@mcri.edu.au (R.F.G.); 3 Department of Paediatrics, University of Melbourne, Parkville 3052, Australia; 4 Newborn Screening Laboratory, Department of Biochemical Genetics, Genetics & Molecular Pathology Directorate, SA Pathology, Women’s & Children’s Hospital, North Adelaide 5006, Australia; kate.coleman@sa.gov.au; 5 Newborn Screening Unit, Chemical Pathology, Pathology Queensland, Royal Brisbane and Womens Hospital, Herston 4029, Australia; gabrielle.crisp@health.qld.gov.au; 6 New South Wales Newborn Screening Programme, The Children’s Hospital at Westmead, Sydney 2145, Australia; enzo.ranieri@health.nsw.gov.au; 7 National Newborn Screening Laboratory, LabPlus, Health New Zealand Te Whatu Ora, Auckland 1023, New Zealand; mark.dehora@tewhatuora.govt.nz (M.d.H.); diannew@adhb.govt.nz (D.W.); nheather@adhb.govt.nz (N.H.); 8 Liggins Institute, University of Auckland, Auckland 1023, New Zealand

**Keywords:** newborn bloodspot screening, residual use, consent, test validation

## Abstract

Newborn screening (NBS) bloodspots are primarily used to test for a defined panel of conditions, yet screening expansion has necessitated the implementation of new tests. Integral to test implementation across all clinical laboratories is the need to evaluate the method with clinical samples, and a common secondary use of residual bloodspots, which are samples that remain following conventional screening, is validating new testing methods by establishing “normal” analyte concentrations and setting action decision limits for NBS protocols prior to implementation. Analysis of de-identified residual bloodspots may potentially reveal an infant with a treatable condition, and re-identification and clinical notification of the infant may then be prudent. However, consent to test residual samples for conditions under implementation may not have been obtained. This ethical dilemma risks the inclusion of residual bloodspots in test development being declined by over-arching NBS authorities. Consequently, the Human Genetics Society of Australasia (HGSA) NBS Committee issued a survey to the Australasian NBS laboratories regarding the current practices of the use of residual bloodspots for new test implementation. The questionnaire revealed a consistent requirement for residual NBS bloodspot analysis to determine reference ranges and screening cutoffs for biochemical variants. If using de-identified residual samples as a component of test validation, re-identification of infants with out-of-range results for clinical referral was considered ethically justified for potentially treatable conditions. The survey results will be used to develop a consensus approach in the region that is both ethically and scientifically valid.

## 1. Introduction

Newborn bloodspot screening (NBS) was established in the late 1960s throughout Australia and New Zealand (collectively referred to as Australasia) with five laboratories in Australia and a single national NBS laboratory in New Zealand performing screening for approximately 350,000 newborns annually with over 98% coverage. The screening programs operate at no cost to the family and test for a consented scope of conditions, as defined by the relevant Government Health Department. Lists of target disorders are available on regularly updated publicly available websites [1,2,3].

In 2023, the Australian Government Department of Health, Disability and Ageing reviewed the scope of conditions of each Australian NBS program and a gap analysis resulted in the addition of several conditions to achieve consistent scope across all programs [1,3]. In order to achieve consistency across Australia, all programs were required to include screening for remethylation defects and guanidinoacetate methyltransferase deficiency, and one laboratory was required to include screening for galactosaemia (where clinical diagnosis was previously considered sufficient) [1]. A further gap analysis involving the Californian NBS program was performed and each condition was individually assessed for screening in Australia [4]. The addition of a further four target conditions was agreed by a formal process under the Australian Government Department of Health, Disability and Ageing for implementation across all programs; these conditions were tyrosinaemia type I, biotinidase deficiency, X-linked adrenoleukodystrophy (X-ALD) and sickle cell disease [1]. New Zealand is undergoing similar expansion with recent implementation of NBS for spinal muscular atrophy (SMA) and consideration of X-ALD.

Implementation of multiple new NBS conditions within a short time frame is challenging for NBS programs. Resources external to the laboratory are required, including development of screening information for health professionals, parental information for informed NBS consent, and development of clinical referral pathways. Within the laboratory, test development includes method selection, equipment selection and purchase, employment of scientific and technical staff, and appropriate training prior to obtaining accreditation for the new tests [5].

Integral to test implementation across all clinical laboratories is the need to evaluate the method with clinical samples. Method evaluation in clinical laboratories is typically performed using anonymised samples, and abnormal test results are not reported [6]. However, Australasian clinicians have expressed a concern about this approach when implementing newborn screening tests, given the inability to identify an infant with a probable treatable disorder from within de-identified residual NBS sample cohorts.

Newborn screening markers include biochemical variables (e.g., enzyme assays or metabolite levels) and genomic markers (e.g., the presence or absence of a disease-causing genetic variant). As genomic markers remain stable over a person’s lifetime, tests can potentially be validated using bloodspots derived from non-infant sources. One Australasian screening laboratory was not given approval by their oversight committee to use residual bloodspot samples in the development of NBS for spinal muscular atrophy (SMA). SMA NBS is a genetic-based test with a specific marker of homozygous deletion of exon 7 in the *SMN1* gene. Development and validation of NBS was performed using quality assurance (QA) and previously diagnosed case–control samples.

In contrast, biochemical newborn screening markers are typically continuous and vary depending on age. Whilst the determination of biochemical assay characteristics (such as reproducibility, linearity) can be done utilising bloodspot samples derived from adults, the determination of age-dependent reference ranges and screening cutoffs necessitates testing bloodspot samples derived from newborns. Alternatives to using residual screening samples during validation include “live testing” screening thresholds derived from other screening laboratories, commercial kits, or post-analytical digital tools. One possible live testing scenario could be reporting only disorder-probable results at the time they are obtained, as very abnormal results are unlikely to be impacted by issues such as standardisation, and holding borderline results for a short period whilst the laboratory gains experience of ranges, and until definitive decision limits are determined.

Despite these possibilities, the utility of using residual bloodspot samples for newborn screening quality assurance and test development is well recognised [7,8,9,10,11,12] and is governed by local policy statements within Australia and New Zealand [13,14,15]. Residual NBS bloodspots may be analysed as untraceable anonymised samples, or more commonly as de-identified samples, meaning that essential information such as gestational age, birth weight, and sex are retained as required for result interpretation. The use of older archival samples, where available, minimises the risk of revealing an unrecognised affected case when developing tests for conditions that present in the neonatal period or in early childhood. However, for later-onset conditions, such as X-ALD and lysosomal storage conditions, there are still risks related to the identification of cases that are yet to present. Furthermore, many biochemical markers are unstable upon long-term storage at ambient temperature conditions [16] and real-time analysis is therefore required for determination of reliable decision limits.

We aimed to seek a regional consensus for an ethically and scientifically valid process of validating tests for new target disorders in newborn bloodspot screening. The HGSA NBS Committee surveyed six Australasian NBS laboratories about their current practices in evaluating new NBS tests mandated through screening expansion, and how they balance the potential dilemma of uncovering an unexpected result that could indicate a treatable condition.

## 2. Materials and Methods

### 2.1. Survey Scenario

The six Australasian laboratories were presented with a method evaluation scenario to survey NBS test development practices:

“A new condition has been mandated for addition to the newborn screening panel and the laboratory has selected a commercially available IVD kit for screening. The newborn screening parent information sheet states that residual material can be used to improve the screening program, method development and validation (including method validation). However, The NBS oversight team refused permission to use de-identified bloodspots as this would potentially jeopardise the health of any baby whose spot gave an out-of-range result. Permission to use identified residual NBS samples was also refused as parents had not initially consented to having this test done.”

### 2.2. Specific Survey Question

Specific questions were asked of each NBS laboratory:

- Is development and validation of a method for a mandated NBS test considered research? If test development is considered research, how is informed consent obtained?
- Which oversight authority gives approval to use residual NBS samples for test development?
- Is informed consent required for conditions under active implementation?
- What quality assurance results are essential in the method validation?
- Are reproducible results required on NBS samples? If so, how many samples are required?
- Are NBS samples used de-identified during test validation?
- The method performs as expected. NBS samples were unable to be tested in validation. Would real-time NBS results be reported?
- Would ISO 15189:2022 [17] accreditation be granted without analysis of normal samples to determine decision limits for referral?

## 3. Results

The survey responses from the six NBS laboratories were collated and compared. These were generally consistent. Some responses were appended with further information. The collated responses were de-identified and are presented in Table 1.

### Survey Response Summary

- Evaluation of a mandated NBS method for program development is not considered to be a research activity. Two programs collect consent for de-identified research independent of NBS method development.
- Approval from the NBS program oversight committee is required. Local ethics approval is also required by some programs.
- Specific informed consent is not required and several programs state that NBS samples may be used for test development. Program protocols allow use of NBS samples for test development.
- Analysis of internal and external quality assurance samples and confirmed case-controls is required.
- NBS tests producing continuum result data require analysis of a large number of NBS samples to determine decision limits for infant referral and test validation. The number of samples is dependent on the quality of the data and statistical characteristics. Data from genetic tests based on variant detection is dichotomous and test validation can be accomplished with minimal or no infant samples.
- NBS samples are analysed as de-identified samples. However, the consensus view was that out-of-range results require re-identification and clinical referral.
- NBS laboratories are accredited to ISO 15189:2022. This standard requires examination of normal data and relevant biological characteristics to establish decision limits and accreditation may not be granted without data from residual bloodspot samples.

## 4. Discussion

The Australasian NBS programs have recently undergone rapid expansion with the inclusion of additional mandated disorders and tests within short implementation timeframes [1]. Development of additional screening tests for quality improvement is a recognised acceptable use of residual bloodspot samples [7,8,9,10,11,12,13,14,15]. Establishing analytical decision limits for NBS tests with continuum data, such as metabolite concentration or enzyme activity, requires testing and statistical analysis of data from large numbers of NBS samples. This is essential to meet established laboratory standards for test validation of an on-going population screening program. As such, the purpose of using NBS samples in the evaluation process of mandated tests for additional conditions is for service development rather than research, which aims to generate new, generalisable knowledge.

Prior to implementation, an NBS test requires analysis at scale to ensure all aspects of the screening process are reliable. This includes laboratory testing with typical work-loads, data export and import into laboratory information management systems and reporting mechanisms and to achieve ISO 15189:2022 accreditation. NBS test analyte data exists as continuum data with metabolite or enzyme-based tests with data that is often skewed with infrequent outliers and extremes requiring large data sets to establish the characteristics and to reliably define the clinical decision referral limits. In contrast, NBS condition testing using DNA-based analysis produces binary data with the presence or absence of a genetic variation (e.g., homozygous deletion of exon 7 in the *SMN1* gene for NBS for SMA), and validation of this type of test may be accomplished using quality control and case–control samples.

Our survey revealed that all Australasian laboratories utilise residual NBS samples for test implementation. Jurisdictional practice includes residual de-identified NBS samples in the final stages of development, once acceptable technical validation has been achieved and with the approval of the clinical treating team. The instability of some NBS analytes (biochemical markers and enzymes) requires use of residual NBS samples in real time during test validation. Discussions with clinicians revealed a concern about potential out-of-range results and the detection of potentially treatable conditions, necessitating the need for re-identification. The recent Australian NBS expansion into testing for conditions that have later-onset diseases, such as X-ALD, may reveal archival samples that have out-of-range results which creates an ethical dilemma with clinicians requesting that these individuals are referred for follow-up.

Our survey also revealed some inconsistent practices across jurisdictions: (a) whether local ethical approval was required for the development of new tests, (b) publicly available information about the use of samples for the development of new tests and (c) consent processes for making samples available for de-identified research. Some of these have a significant impact on the ability to implement new tests and represent an opportunity for future harmonisation in order to streamline the introduction of new NBS tests.

Whilst newborn bloodspot screening is mandatory in other parts of the world, screening participation in the Australasian NBS programs occurs with parental consent on the scope of testing. Screening uptake is therefore reliant on maintaining a high level of public trust and support from health care providers. NBS tests under development are beyond the scope listed on jurisdictional websites and consumer information at that time. This places the NBS laboratory in the difficult position of developing an NBS test for a mandated potentially treatable condition that may not yet be listed as a target disorder on consent materials and jurisdictional websites. However, disorders mandated for implementation in our region have been through an extensive process of review by an expert panel of clinicians and public health representatives and can be assumed to be serious, treatable health conditions. In this respect, disclosure of any disorder-probable results prior to formal implementation can be considered as acting in the best interests of the infant. Whilst Australasian parents have not been asked their views on test implementation, analogous research in genomic newborn screening demonstrates that parents would expect to receive information that is pertinent to their newborn [18,19].

In New Zealand, NBS samples are considered to belong to the person from whom they were collected [14]. Ownership of NBS samples in Australia is more complex and includes ownership of the sample card and of the bloodspot sample. NBS sample ownership remains incompletely resolved and a legal framework reflecting a balance between the rights of parents and children and developing and maintaining a high-quality NBS program is yet to evolve [20]. The Royal College of Pathologists of Australasia (RCPA) suggest that a concept of “custodianship” is preferable [21]. The idea that a “body part,” once removed for testing, belongs to no one is contentious [20].

The Australian Government Department of Health, Disability and Ageing lists the target and non-target conditions screened in the five Australian programs. Nationally agreed NBS conditions agreed as targets and under implementation are also listed [1]. To maintain the high degree of public trust in Australian NBS programs, statements on storage, consent and further use of the residual NBS samples must be clear and should ideally be nationally consistent. The delineation of secondary uses of residual bloodspot samples for quality improvement and NBS test development for the benefit of the population, as distinct to research use, may not be apparent to the general public. In a recent review of 654 published studies on the secondary use of residual bloodspots for quality improvement or research, information on whether consent was obtained or whether non-consented de-identified bloodspots were used was inconsistently and incompletely reported [10].

Clarity on the further use of residual bloodspots for nationally agreed and mandated testing is essential. To this end, one Australasian NBS program has enlarged the parental consent statement to encompass these nationally approved and mandated conditions listed and under active implementation [22]. If the NBS results affect the health of the baby, the infant will be referred and parents will be contacted. This statement is similar to that issued by NHS England [11]. Residual NBS samples are included in the development phase only when the NBS test is shown to be technically reliable, quality assurance and case–control samples are concordant, the test is close to validation and testing is in conjunction and agreement with the clinical referral team.

## 5. Conclusions

The Australasian NBS programs are undergoing rapid expansion with the implementation of several mandated NBS conditions within a short time period and under active concurrent test development. A survey conducted by the HGSA NBS Committee on test development processes showed inconsistencies in laboratories’ use of local ethical approval and the publicly available information on the use of samples for test development. The survey also showed that the laboratories consistently considered test development and validation to be a quality and program improvement process and not research. Test implementation required the essential use of residual bloodspots for validation and specific consent was not required. In test development, bloodspots were analysed in de-identified form; however, infants with out-of-range results from a potentially treatable condition would require re-identification and infant referral. The use of archival samples for those conditions that present in the neonatal period will not be impacted to the same degree as those conditions that present in childhood. Biochemical- and enzymatic-based analytes that are unstable under long-term storage will require real-time analysis to establish suitable cut-offs and action limits. Consent statements in one program have been expanded to include conditions agreed as target conditions and listed as under implementation. This approach may be considered as a way forward for other screening laboratories. Overall, national harmonisation of the above issues is desirable to streamline the introduction of new NBS tests and achieve consistency in the information provided to the public and health professionals. These findings may also inform international discussions on governance and consent frameworks for residual newborn screening samples, particularly in settings undergoing rapid panel expansion.

## Figures and Tables

**Table 1:** Survey responses of six Australasian NBS programs.

Criterion	Consistent Responses	Alternative Responses and Notes
Is development and validation of a method for a mandated NBS test considered research? If test development is considered research, how is informed consent obtained?	Six NBS programs: No. Mandated NBS test development is quality improvement. It is not research.	Two NBS programs: A consenting statement and tick box for de-identified research is on the NBS collection card.
2. Which oversight authority gives approval to use residual NBS samples for test development?	Six NBS programs: Require authority by an independent authority, e.g., The NBS program oversight committee, department of health.	Three NBS programs: local ethics approval required.
3. Is informed consent required for conditions under implementation?	Six NBS programs: Informed consent is not required	Three NBS programs: formal statement publicly that samples may be used to develop new tests and methods without specific consent.
4. What quality assurance results are essential in the method validation?	Six NBS programs: Reproducible and consistent results on internal and external quality assurance control samples.	Six NBS programs: Bloodspot samples from clinically confirmed cases should be included.
5. Are reproducible results required on NBS samples? If so, how many samples are required?	Six NBS programs: Consistent NBS results are required for continuum data (metabolite or enzyme) on large numbers of samples, N = 3000 to 35,000	The number of samples is dependent on data quality and statistical characteristics. Validation of dichotomous genetic data may require minimal or no NBS samples.
6. Are NBS samples used de-identified during test validation?	Five NBS programs: Samples are de-identified during validation. Due to ethical concern, out-of-range results are re-identified and reported to the clinical team.	Use of identified samples may conflict with existing consent statements. One NBS program was denied use of NBS samples for test development by decision of NBS oversight committee.
7. The method performs as expected. NBS samples were unable to be tested in validation. Would real-time NBS results be reported?	Three NBS programs: Stated an NBS test would not be implemented without assaying residual NBS samples.	One NBS program: Test implementation required without using residual NBS samples. Two NBS programs: NBS testing would proceed with referral of out-of-range results and withholding normal results. Six NBS programs: Approval required of the appropriate clinical team prior to NBS testing for potentially reportable results.
8. Would ISO 15189:2022 accreditation be granted without analysis of normal samples to determine decision limits for referral?	Five NBS programs: Successful accreditation considered unlikely without testing NBS samples. Age, biological sex and other relevant information must be considered when establishing decision limits	Reference could be made to decision limits reported by other laboratories. One NBS program required to implement without testing NBS samples by decision of NBS oversight committee.

## Data Availability

The original contributions presented in the study are included in the article, further inquiries can be directed to the corresponding author.
